# The methodological quality of animal research in critical care: the public face of science

**DOI:** 10.1186/s13613-014-0026-8

**Published:** 2014-07-29

**Authors:** Meredith Bara, Ari R Joffe

**Affiliations:** 1 Faculty of Medicine and Dentistry, University of Alberta, Edmonton T6G 2R3, Alberta, Canada; 2 Department of Pediatrics, 4-546 Edmonton Clinic Health Academy, University of Alberta, 11405 87 Avenue, Edmonton T6G 1C9, Alberta, Canada

**Keywords:** Animal research, Critical care, Intensive care, Methodology

## Abstract

**Background:**

Animal research (AR) findings often do not translate to humans; one potential reason is the poor methodological quality of AR. We aimed to determine this quality of AR reported in critical care journals.

**Methods:**

All AR published from January to June 2012 in three high-impact critical care journals were reviewed. A case report form and instruction manual with clear definitions were created, based on published recommendations, including the ARRIVE guidelines. Data were analyzed with descriptive statistics.

**Results:**

Seventy-seven AR publications were reviewed. Our primary outcome (animal strain, sex, and weight or age described) was reported in 52 (68%; 95% confidence interval, 56% to 77%). Of the 77 publications, 47 (61%) reported randomization; of these, 3 (6%) reported allocation concealment, and 1 (2%) the randomization procedure. Of the 77 publications, 31 (40%) reported some type of blinding; of these, disease induction (2, 7%), intervention (7, 23%), and/or subjective outcomes (17, 55%) were blinded. A sample size calculation was reported in 4/77 (5%). Animal numbers were missing in the Methods section in 16 (21%) publications; when stated, the median was 32 (range 6 to 320; interquartile range, 21 to 70). Extra animals used were mentioned in the Results section in 31 (40%) publications; this number was unclear in 23 (74%), and >100 for 12 (16%). When reporting most outcomes, numbers with denominators were given in 35 (45%), with no unaccounted numbers in 24 (31%), and no animals excluded from analysis in 20 (26%). Most (49, 64%) studies reported >40, and another 19 (25%) reported 21 to 40 statistical comparisons. Internal validity limitations were discussed in 7 (9%), and external validity (to humans) discussed in 71 (92%), most with no (30, 42%) or only a vague (9, 13%) limitation to this external validity mentioned.

**Conclusions:**

The reported methodological quality of AR was poor. Unless the quality of AR significantly improves, the practice may be in serious jeopardy of losing public support.

## Background

Translation of biomedical animal research (AR) findings to humans has been disappointing [1],[2]. There are two main possible reasons for this. First, animals are complex biological systems; their nonlinear dynamics and responses are extremely sensitive to initial conditions [3],[4]. Despite superficial physiologic and genetic similarity between species, it may not be that responses to similar perturbations or disease will be relevantly similar. Second, the methodological quality of AR may be poor, causing misleading results [5]–[9]. A third possibility that attempts at translation are made prematurely (or badly), before an intervention is well understood, seems less likely to account for failed translation of the many very promising preclinical interventions studied in multiple clinical trials.

The claims made above are supported by much empirical literature. First, the poor translation rate of AR to human medicine has been found in critical care, for example, in the fields of sepsis [10]–[12], traumatic brain injury [13], resuscitation [14], and spinal cord injury [15]. This has also been found in other highly researched medical fields such as stroke [7], asthma [16], cancer [17], and pharmaceutical drug development [18]. Second, poor methodological quality of AR has been reported in many publications over the past four decades [5]–[9],[19]–[26]. The lack of randomization, allocation concealment, blinding, primary outcome and sample size calculation, as well as multiple statistical testing, and publication bias have been assumed to account for the poor translation of AR to human medicine [3],[6],[8],[27]. The ARRIVE guidelines [28], supported by many high-impact journals, and other national guidelines [29]–[31], suggest inclusion in publications of these methodological factors that are found to be poorly reported. Third, a growing literature suggests that responses to interventions are different in different species due to in principle differences in initial conditions of complex systems (the organism) resulting in different genomic (and hence functional) outcomes [3],[4],[32]–[37].

For example, no novel therapy based on AR has been successful in the treatment of sepsis in humans [10]–[12]. This may be explained by the finding that the genomic responses to different acute inflammatory stresses, including trauma, burns, and endotoxemia/sepsis are highly similar in humans; however, these responses are not reproduced in mouse models [32]. Among genes changed significantly in humans in these diseases, ‘the murine orthologs are close to random in matching their human counterparts’ [32]. Indeed, lethal toxicity to bacterial lipopolysaccharide varies almost 10,000-fold in different species [38]. Interestingly, of 120 essential human genes with mouse orthologs, 17 (22.5%) were nonessential in mice, suggesting that ‘it is possible that mouse models of a large number of human diseases will not yield sufficiently accurate information [36]’. Compatible with this, the ENCODE project suggests that over 80% of the genome is functionally important for gene expression; it is likely there are ‘critical sequence changes in the newly identified regulatory elements that drive functional differences between humans and other species [37]’. This may also explain ‘the specific organ biology [from lineage-specific gene expression switches] of various mammals [35]’. These, and other similar findings, suggest that a systems biology approach to the nonlinear complex chaotic dynamics of mammalian organisms in which responses are extremely sensitive to initial conditions (the genome and its epigenetic regulatory mechanisms) explains the lack of translation. By this explanation, in principle, AR findings will not predict human responses.

One step in settling this debate in critical care AR is to determine the most current methodological quality of the relevant AR. To address this, we aimed to determine the reported methodological quality in critical care AR published in the year 2012. We find that the reported methodological quality of AR published in three high-impact critical care journals during 6 months of the year 2012 was poor, potentially contributing to the poor translation rate to human medicine.

## Methods

### Ethics statement

The University of Alberta Health Research Ethics Board waived the requirement for review because the study involved only publicly available data.

We reviewed all consecutive AR published in three prominent critical care journals (*Critical Care Medicine*, *Intensive Care Medicine*, and *American Journal of Respiratory and Critical Care Medicine*) during 6 months of the year 2012 to determine the reporting of *a priori*-determined methodological quality factors. There were no restrictions other than that the study reported an AR experiment, defined as a procedure for collecting scientific data on the response to an intervention in a systematic way to maximize the chance of answering a question correctly or to provide material for the generation of new hypotheses [26]. Both authors hand-searched and screened the titles and abstracts of all publications in the three journals over the 6 months, and if possibly a report of an AR experiment, the full text was reviewed. If there was any doubt about the inclusion, this was discussed among the two authors to achieve consensus. A data collection form and instruction manual (see Additional files 1 and 2) were created based on published Canadian, US, and UK recommendations for reporting AR [28]–[31]. These guidelines were used as they are comprehensive, well referenced, readily available, and based upon literature review. For example, the ARRIVE guidelines were developed to improve the quality of reporting AR and are endorsed by over 100 journals from all over the world [28]. Data were obtained for factors important to methodological quality. We also reviewed these publications to determine the reporting of *a priori*-determined ethical quality factors and have reported this elsewhere [39]. From inception, we considered the ethical and methodological quality as separate issues and decided that reporting them separately was needed to adequately report each issue and discuss its implications.

The form was completed for all consecutive critical care AR (using mammals) publications (including all supplemental files online) from January to June 2012 in the three critical care journals. Both authors independently completed forms for the first 25 papers, discussing the data after every fifth form until consistent agreement was obtained. Thereafter, one author completed forms on all papers, and the other author independently did so for every fourth paper (with discussion of the data to maintain consistent agreement) and for any data considered uncertain (with discussion until consensus). The instruction manual made clear definitions for all data collection; for example, a sample size calculation was defined as describing, for the primary outcome, a *p* value (alpha), power (1-beta), and minimally important difference (the difference between groups that the study is powered to detect).

### Statistics

This is an exploratory descriptive study. Data are presented using descriptive statistics and were analyzed using SPSS. The primary outcome was pre-specified as the composite of reporting the three animal characteristics of strain, sex, and weight or age. In the largest previous survey of AR (not limited to critical care, reviewing publications from 1999 to 2005, and leading to the ARRIVE guidelines), this composite outcome was the primary outcome and reported in only 59% (159/271) of publications [5]. These variables are important to report to allow replication of AR, and poor replicability of AR results has been a major problem in recent literature [40],[41]. Our study was designed to determine a reasonable 95% confidence interval (CI) for this primary outcome. Assuming a similar reporting rate of 59% to have an adjusted Wald 95% CI of ±11%, we pre-specified a sample size of 75 publications. Pre-defined subgroups by journal, sepsis model, and animal age (neonate, juvenile, adult) were compared using the Chi-square statistic, with statistical significance accepted at *p* < 0.05, without correction for multiple comparisons. *Post hoc* we identified another subgroup of rodent/rabbit versus nonrodent/nonrabbit models to determine whether more advanced species had improved attention to the methodological quality of AR. We also determined three *post hoc* composite outcomes: (a) reporting of randomization and any blinding and numbers given with denominators for most outcomes; (b) reporting of the criteria mentioned in (a) and also meeting our pre-defined primary outcome of animal descriptors; and (c) reporting of the criteria mentioned in (b) and also having reported allocation concealment, blinding of subjective outcomes, and no unaccounted animal numbers for most outcomes.

## Results

Results from the review of 77 AR publications (Additional file 3) in three critical care journals are in Tables 1, 2, 3, 4 and 5. For ease of reporting the results, we divide the reporting into the specific sections of the publications; however, if the variable of interest was reported anywhere in the manuscript, we considered it as having been reported.

**Table 1:** **Reported methodological quality of animal research published in three critical care journals in 2012:Methodssection**

**Criterion**	**Number of 77 publications meeting criterion,** ***n*** **(%) [95% confidence interval]**
Randomization reported	47 (61%) [50%, 71%]
Allocation concealment mentioned	3 (6% of 47) [2%, 18%]
Randomization procedure described	1 (2% of 47) [<1%, 12%]
Reported blinding of any type mentioned below	31 (40%) [30%, 51%]
Disease induction	14 (45% of 31) [29%, 62%]
Intervention	7 (23% of 31) [11%, 40%]
Subjective outcomes	17 (55% of 31) [38%, 71%]
Primary outcome specified	5 (7%) [2%, 15%]
Sample size calculation reported	4 (5%) [2%, 13%]
More than 10 secondary outcomes specified	74 (96%) [89%, 99%]
Eligibility criteria for animals stated	4 (5%) [2%, 13%]
Acclimation/habituation prior to experiments stated	6 (8%) [3%, 16%]
Staff (number or training) performing experiment described	1 (1%) [<1%, 8%]
Animal numbers stated in methods section	61 (79%) [69%, 87%]
Animal numbers (when stated)	Median 32 (range 6 to 320; IQR 21 to 70)
Sepsis model: with any supportive therapy mentioned^a^	12 (44% of 27) [28%, 63%]

^a^Sepsis supportive therapies were fluids, 11 (41% of 27) and antibiotics, 4 (15% of 27). Another 1 (4% of 27) had animals with the co-morbid illness of trauma. The intervention was given only pre-sepsis induction in 7 (26% of 27). IQR, interquartile range.

**Table 2:** **Reported methodological quality of animal research published in three critical care journals in 2012:Resultssection**

**Criterion**	**Number of 77 publications meeting criterion,** ***n*** **(%) [95% confidence interval]**
Animal descriptions reported	
Strain	67 (87%) [78%, 93%]
Sex	59 (77%) [66%, 85%]
Age	29 (38%) [28%, 49%]
Developmental stage	27 (35%) [25%, 46%]
Developmental stage when given	Neonate 5, juvenile 1, adult 21
Weight	60 (78%) [67%, 86%]
Animal source^a^	33 (43%) [32%, 54%]
Baseline characteristics of treatment groups described^b^	23 (31%) [21%, 41%]
Outcomes reported	
Number of animals in largest treatment group 10 or less	61 (79%) [69%, 87%]
Extra animals used in the results (that were not stated in methods)	31 (40%) [30%, 51%]
Number of extra animals unclear	23 (74% of 31) [57%, 87%]
Number of extra animals >100	12 (39% of 31) [24%, 56%]
Numbers with denominators given when reporting the majority of outcomes^c^	35 (45%) [35%, 57%]
No unaccounted animal numbers for the majority of outcomes	24 (31%) [22%, 42%]
No animals excluded from analysis for the majority of outcomes^d^	20 (26%) [17%, 37%]
Animal numbers provided in the majority of tables/graphs	46 (60%) [49%, 70%]
Number of statistical comparisons reported	
>40	49 (64%) [52%, 74%]
21 to 40	19 (25%) [16%, 35%]
5 to 20	9 (12%) [6%, 21%]
Any negative outcome reported in results^e^	15 (20%) [12%, 30%]
If applicable, toxicity or lack of toxicity to animals was mentioned	11 (22% of 49) [13%, 36%]
No *post hoc* outcomes analyzed^f^	40 (52%) [41%, 63%]

^a^Animal sources were commercial, 29 (85% of 34) and local, 5 (15% of 34); ^b^baseline characteristics described were at least two demographic variables 1 (4% of 24) and at least two physiologic variables 19 (79% of 24); ^c^for the AR articles’ primary outcome (specified in five studies): numbers with denominators reported for 3 (60% of 5), no unaccounted numbers for 3 (60% of 5), and numbers in tables/graphs provided in 3 (60% of 5); ^d^when some animals were excluded from most analyses, the number excluded (10, 18% of 57) and reasons (11, 19% of 57) were reported infrequently. For the AR articles’ primary outcome, an intention to treat analysis was used for 2 (40% of 5); ^e^for the AR articles’ primary outcome, a negative result was reported in 0 (0% of 5); ^f^number of *post hoc* outcomes: none in 40 (52% of 77), <5 in 25 (32% of 77), 5 to 10 in 8 (10% of 77), and >10 in 4 (5% of 77).

**Table 3:** **Reported methodological quality of animal research published in three critical care journals in 2012:Discussionsection**

**Criterion**	**Number of 77 publications meeting criterion,** ***n*** **(%) [95% confidence interval]**
Internal validity limitations discussed^a^	7 (9%) [4%, 18%]
External validity (to humans) discussed	71 (92%) [84%, 97%]
When discussed, no limitation to external validity (to humans) mentioned	30 (42% of 71) [31%, 54%]
When discussed, only a vague limitation to external validity mentioned	9 (13% of 71) [7%, 23%]

^a^Internal validity limitations: sample size in 5, methodological bias in 3, and multiple statistical comparisons in 1.

**Table 4:** Reported methodological quality of animal research published in three critical care journals in 2012: primary and composite outcomes

**Criterion**	**Number of 77 publications meeting criterion,** ***n*** **(%) [95% confidence interval]**
This study’s pre-defined primary outcome	
Animal strain, sex, and weight or age described	52 (68%) [56%, 77%]
Composite quality outcomes	
Reported randomization and any blinding, and numbers given with denominators for the majority of outcomes	14 (18%) [11%, 28%]
Criteria above and meeting this study’s pre-defined primary outcome of animal descriptors	8 (10%) [5%, 19%]
Criteria above and reporting of allocation concealment, blinding of subjective outcomes, and no unaccounted animal numbers for the majority of outcomes	0 (0%) [0%, 4%]

**Table 5:** Methodological quality of animal research published in three critical care journals: rodent/rabbit versus nonrodent/nonrabbit subgroup

**Criterion**	**Number of publications meeting criterion;** ***n*** **(%) [95% CI]**		
	**Rodent/rabbit (*n*** **= 54)**	**Nonrodent/nonrabbit (*n*** **= 23)**	***p*** **value**
This study’s pre-defined primary outcome			
Animal strain, sex, and weight or age described	45 (83%) [71%, 91%]	7 (30%) [15%, 51%]	<0.001
Methods			
Animal numbers stated in methods	35 (65%) [51%, 76%]	21 (91%) [72%, 99%]	0.049
Reporting randomization	25 (46%) [34%, 59%]	22 (96%) [77%, >99%]	<0.001
Results: animal descriptions reported			
Sex	48 (89%) [77%, 95%]	11 (48%) [29%, 67%]	<0.001
Weight	38 (70%) [57%, 81%]	22 (96%) [77%, >99%]	0.011
Source	30 (56%) [42%, 68%]	3 (13%) [4%, 33%]	<0.001
Results: outcomes reported			
Extra animals used in the results (that were not stated in methods)	27 (50%) [37%, 63%]	4 (17%) [6%, 38%]	0.007
Animal numbers in the majority of tables and graphs	37 (69%) [55%, 79%]	9 (39%) [22%, 59%]	0.016
Baseline characteristics of treatment groups described	9 (17%) [9%, 29%]	15 (65%) [45%, 81%]	<0.001
Discussion			
Limitation to external validity (to humans) mentioned	16 (33%) [19%, 43%]	16 (70%) [49%, 85%]	0.002
Composite quality outcomes			
Reporting randomization and any blinding, and numbers given with denominators for the majority of outcomes	10 (19%) [10%, 31%]	4 (17%) [6%, 38%]	ns
Criteria above and meeting this study’s pre-defined primary outcome	8 (15%) [7%, 27%]	0 (0%) [0%, 13%]	ns

Animals in the publications were nonrodent/nonrabbit- baboon (1), dog (3), pig (17), sheep (2); rodent/rabbit- mouse (17), rabbit (5), and rat (32). There were no statistically significant differences between these subgroups in any of the other methodological criteria shown in Tables 1, 2, 3 and 4. ns, not significant.

### Our primary outcome (animal descriptors)

Animal strain, sex, and weight or age were reported in 52 (68%; 95% CI, 56% to 77%) of publications.

### Reporting in the methods section

In the 47 (61%) studies reporting randomization, the randomization method (1, 2%) and allocation concealment (3, 6%) were rarely reported (Table 1). A minority of studies reported blinding (31, 40%), and this included for subjective outcomes (17/31, 55%). Reporting a sample size calculation (4, 5%) and specifying a primary outcome (5, 7%) were almost never done. Animal numbers were often not reported; animal numbers were given in 61 (70%) and when stated were a median of *n* = 32 (interquartile range 21 to 70, range 6 to 320). Eligibility criteria (inclusion and exclusion) for animals were reported in only 4 (5%).

### Reporting in the results section (animal descriptions)

Species, strain, and sex were usually described (77, 100%; 67, 87%; and 59, 77%, respectively); however, age (29, 38%), developmental stage (27, 35%), and description of baseline characteristics in treatment groups (23, 31%) were often missing (Table 2).

### Reporting in the results section (outcomes)

Extra (31, 40%), unaccounted for (53, 69%), and excluded animals (57, 74%) were common. Extra animals were defined as follows: the number of animals used in the Results section is different from and higher than that stated in Methods section. Unaccounted for animals was defined as follows: the number of animals used in most analyses was lesser than the number given in the Methods section for unclear reasons. Excluded animals were defined as follows: animals that were stated to be used in the experiments were excluded from the majority of analyses. Outcomes were often reported without denominators in text and tables/graphs (31, 40%). Most studies performed >40 statistical analyses (49, 64%), often of *post hoc* outcomes (37, 48%), with mention of negative (15, 20%) or toxicity (applicable if a drug was being studied; 11/49, 22%) outcomes uncommon (Table 2).

### The discussion section

Internal validity limitations were rarely discussed (7, 9%). External validity (to humans) was mentioned in 71 (92%); however, limitations to this external validity were often not mentioned (32/71, 45%) (Table 3).

### Composite outcomes

Fourteen (18%) met the composite outcome of reporting any randomization, any blinding, and numbers given with denominators for most outcomes; only 8 (10%) met the composite outcome of the aforementioned criteria and adding meeting our primary outcome of animal descriptors (Table 4).

### Funding sources

Funding source was reported for 69 (90%) of the publications. Most studies were funded using public dollars, either from government (51/69, 74%) and/or foundation/charity (34/69, 49%); industry funding was uncommon (11/69, 16%).

### Subgroups

Sepsis models (*n* = 27) and studies in the higher-impact journal were lower in quality (less often reporting randomization, stating animal numbers in the Methods section, and reporting animal weight; all *p* ≤ 0.004). Adult animal studies more often reported sex and our primary outcome of animal descriptors (*p* < 0.01). The *post hoc* subgroup of nonrodent/nonrabbit (vs. rodent/rabbit) AR showed few differences in quality practices (Table 5). The nonrodent/nonrabbit publications more often compared baseline characteristics of treatment groups and mentioned limitation to external validity; they less often had extra animals used in the results that were not mentioned in the Methods. However, they have more often missing animal numbers in most tables/graphs and did not have better reporting of the composite quality outcomes.

## Discussion

The reported methodological quality of AR in three high-impact critical care journals during 6 months of 2012 was poor. This is important for several reasons. First, poor attention to reporting optimal methodology in AR confounds the interpretation and extrapolation of experimental results [5],[27]–[31]. Thus, attention to reporting methodological quality is necessary to performing reliable quality research. Second, the interests of sentient animals in avoiding harm ought to be given more consideration in the reporting of AR [42],[43]. The ethical justification of biomedical AR that can harm animals (by any associated distress and death) usually includes reference to its necessity for producing large benefits to human medicine [1],[2],[44]. Thus, this ethical justification of AR assumes the reporting of high-quality research necessary to produce these benefits [1],[2],[6],[45]. Third, attempted translation to humans from methodologically weak AR unnecessarily puts humans at risk and wastes scarce research resources. Fourth, these publications are, arguably, the public face of science using mostly public funds. Unless the methodological quality of AR reporting improves, AR is at risk of losing public support. Recent surveys suggest public support for AR is based on the assumption that attention to the 3Rs (refinement, reduction, replacement) is a priority; public support for AR is far from universal and may be tenuous [46]–[48].

We reported separately the ethical quality of the same AR publications examined here and found that it was poor [39]. Few publications (5/71, 7%) reported monitoring the level of anesthesia during invasive procedures, even when muscle paralytics were used (2/12, 17%). Few publications reported monitoring (2/49, 4%) or treatment (7/49, 14%) of expected pain. When euthanasia was used, the method was reported for 38/65 (59%) of publications; in these, euthanasia was reported to be of an acceptable or justified conditionally acceptable method for the species in 16/38 (42%). [39]. This adds to the problem of translation from AR to humans because pain and distress cause changes in physiology, immunology, and behavior that confound interpretation and extrapolation of experimental results [49],[50].

Limitations of this study include the limited sample size of publications reviewed, the limited scope to critical care AR, and the low power to detect differences between subgroups particularly given multiple comparisons. We did not determine inter-rater reliability of data extraction, and it is possible that our methods of ensuring consistent agreement were insufficient. Finally, our composite outcomes were defined *post hoc*, and although they give a general idea of the way AR reported several quality criteria in the same study, they should be interpreted with caution. Nonetheless, this study is the first to focus on AR in critical care and reviewed a reasonable number of consecutive publications in three high-impact critical care journals using an objective data collection form and instruction manual. Whether our findings from this critical care AR cohort generalize to most AR is unknown; however, we believe this is likely because many others have reported similar findings in other AR fields in the past [5]–[9],[19]–[25].

Another limitation is that we only describe reporting of the quality items; it may very well be that what was not reported was actually done. Thus, it is possible that the methodological quality of the AR was good, and only the reporting was poor. This explanation is problematic for several reasons. First, many of these quality items might have been expected to be reported if they were indeed performed. For example, if a sample size calculation for a pre-specified primary outcome, including a *p* value, power, and minimally important difference, was calculated, the authors would plausibly be expected to report this knowing that it would markedly improve the quality of their experimental result. Optimal methods of randomization, allocation concealment, and blinding may be difficult, time consuming, and expensive to implement, and are known to strengthen the importance and validity of a study; this makes it implausible that these would not be reported if they were done [6],[19]–[22]. Second, many of the quality items we found missing are necessary for readers to adequately evaluate the internal and external validity of the study and to understand and be able to reproduce the methods and results [28]–[31]. For example, the strain, sex, age, weight, source, and baseline characteristics of animals are important potentially confounding variables in a study; understanding research subject numbers and flow are important to understand the methodology and analysis of a study; and multiple statistical testing, particularly with *post hoc* outcomes, weaken any inferences that can be made from study results [27]–[31]. Not reporting this information thus makes the published study findings unreliable, regardless of whether the information was in fact known to the authors. Third, that very few studies discussed internal validity limitations suggests that the authors may not recognize the importance of the methodological factors and may not have incorporated them into their study design.

Poorly reported methodological quality of AR has been reported before [19]–[26]. In fact, the lack of randomization, allocation concealment, blinding, eligibility criteria, primary outcome, and sample size calculation, as well as multiple statistical testing, and publication bias have been assumed to account for the poor translation of AR to human medicine [3],[6],[8],[27],[51]. In both human and animal research, lack of reporting of these items is associated with overestimation of intervention efficacy [19]–[22],[52],[53]. Our findings significantly add to this literature because previous publications have not focused on the entire spectrum of these quality variables, were done before some of the recent guidelines on optimal AR were published, and/or did not focus on critical care AR in particular, as in this study. One other study determined that methodological quality of AR reporting experimental allergic encephalitis models of multiple sclerosis has not improved between 2 years before and 2 years after endorsement of the ARRIVE guidelines [54].

These findings are concerning. The ARRIVE guidelines, supported by many high-impact journals, and other national guidelines, suggest inclusion in publications of the factors that we found to be poorly reported [28]–[31]. Given the generally poor translation rate of AR to human medicine [6]–[8],[13]–[18],[27],[55],[56] (e.g., in the field of sepsis, no novel therapy based on AR has been successful in treatment of sepsis in humans) [10]–[12], researchers should seriously consider whether this is because of lack of sufficient attention to methodological quality, including factors we did not assess in this paper, such as publication bias. This is particularly true because one alternative explanation is that biological differences between species make AR in principle, based on complexity science, unable to predict responses in humans. AR where the experimental question is subject to study solely by reductionism, that is, by examining simple systems at a gross level (for example, discovering the germ theory of disease, that the heart circulates blood, and that the immune system reacts to foreign entities), may translate [57]. However, for the details, such as whether the animal model will accurately predict human response to drugs and disease, complexity science suggests an in principle limitation to AR.

It is true that some findings from AR have translated to humans; one example is the use of lower tidal volumes in acute respiratory distress syndrome (ARDS) to limit ventilator-induced lung injury [58]. This may be because the AR these interventions were based on was of higher quality. However, a retrospective look at interventions that successfully translated does not provide a complete picture of the accuracy for translation of an animal model. For example, a recent review of interventions for ARDS found that only two interventions (low tidal volume and prone positioning) from 93 human trials of over 37 interventions had robust evidence of translation, and one was harmful (HFO) [59]. Even for lower tidal volume, there was a question whether this was beneficial when compared to relatively higher tidal volume that limited airway pressures [59]. A systematic review of the AR relating to VILI to examine the methodological quality of studies, assess publication bias, and determine the association of quality with efficacy would be very informative in this debate.

We note that an improved methodological quality will reduce the flexibility in design, definitions, outcomes, and analytical modes in a study and thus improve the reliability of a reported *p* value (i.e., reduce ‘*p*-hacking’) [60],[61]. However, this will not prevent misinterpretation of the *p* value [62],[63]. Although it is sometimes thought that *p* < 0.05 means that the probability of the null hypothesis is <5%, this is false. For example, in a human trial with equipoise, the prior probability of the null hypothesis is 50%, and a *p* = 0.05 means the probability of the null hypothesis is down to no lower than 13% [64]–[66]. The probability of the null hypothesis depends on its prior probability and the Bayes factor (a measure of the likelihood of the null hypothesis after the study evidence, relative to the likelihood before the study) which can be calculated based on the *p* value [65]. Thus, the *p* value that reduces the probability of the null hypothesis to no less than 5% depends on the prior probability of the null hypothesis: 17% prior probability needs *p* = 0.10, 26% needs *p* = 0.05, 33% needs *p* = 0.03, 60% needs *p* = 0.01, and 92% needs *p* = 0.001 [64],[65]. This has the following implications for AR: the methodological quality must be optimized so that the reported *p* value is robust; studies should be based on external evidence (mechanistic, observational, clinical) that makes the prior probability of the null hypothesis lower that 50%; and if an exploratory study is done (where the null hypothesis is likely), it should be followed by a replication study with the same design and outcome (because the null hypothesis has become less likely) [60]–[65]. The low replication rate of much AR [40],[41] suggests that either these methodological issues are at fault, or, that AR will not translate in principle, based on considerations from complexity science.

We believe that a serious debate about the methodological quality of AR in critical care is urgent. Better attention to, and reporting of, methodological factors in AR can only improve the research quality, ethical quality, and public perception of AR, and improve the safety of humans in translational research. As we reported elsewhere, improved attention to the ethical dimension of AR can only improve these factors as well [39]. Journal editors and reviewers and funding agencies should use their influence to improve quality reporting of AR they publish and support [67],[68]. This includes endorsing and enforcing reporting standards, such as the ARRIVE guidelines, and prioritizing and publishing well-conducted negative studies and replication studies in addition to novel positive studies. Editors and funders hold substantial power to improve the quality of AR and reduce publication bias.

## Conclusions

We found that reported methodological quality of AR in three high-impact critical care journals during 6 months of the year 2012 was poor. These findings warrant the attention of clinicians, researchers, journal editors and reviewers, and funding agencies. Improved attention to the reporting of methodological quality by these groups can only improve AR quality and the public perception of AR.

## Competing interests

The authors declare that they have no competing interests.

## Authors’ contributions

ARJ contributed to conception and design, acquisition of data, analysis and interpretation of data, and drafted the article. MB contributed to design, acquisition of data and interpretation of data, revising the article critically for important intellectual content. ARJ had full access to all the data in the study and takes responsibility for the integrity of the data and the accuracy of the data analysis. ARJ conducted and is responsible for the data analysis. Both authors read and approved the final manuscript.

## Additional files

## Supplementary Material

Additional file 1: **Manual for the Case Report Form for Methodological Quality of animal research study.** This file includes the glossary for all the variables that were abstracted from each animal research study. The definitions used are described in this file. Click here for file

Additional file 2: **Case Report Form for Methodological Quality of animal research study.** This file shows the case report form used to abstract the variables from each animal research study, according to the definitions given in the manual for the case report form. Click here for file

Additional file 3: **The publications included in the Methodological Quality of animal research study.** This file includes the list of the 77 publications reviewed for this study. Click here for file
