# Supplementary material for: The methodological quality of animal research in critical care: the public face of science
Source: Ann Intensive Care. 2014 Jul 29;4:26. doi: 10.1186/s13613-014-0026-8 (PMC4126494; doi:10.1186/s13613-014-0026-8)
Supplement: Additional file 1: — Manual for the Case Report Form for Methodological Quality of animal research study. This file includes the glossary for all the variables that were abstracted from each animal research study. The definitions used are described in this file. [file s13613-014-0026-8-S1.pdf]

Additional File 1: Manual for the Case Report Form for Methodological Quality of animal research study.

Authors: Meredith Bara BSc, and Ari R Joffe MD, FRCPC

Animal: refers to mammals. We include all experiments involving mammals.

Experiment: a procedure for collecting scientific data on the response to an intervention in a systematic way to maximize the chance of answering a question correctly or to provide material for the generation of new hypotheses. It involves some treatment or other manipulation that is under the control of the experimenter, and the aim is to discover whether the treatment is causing a response in the experimental subjects (mammals in this study) and/or to quantify such response. As opposed to observational study that is to find associations between variables that the scientist cannot usually control.

Title: accurate and concise. Is the title misleading in terms of the study design, methodology, or intervention. Examples: incorrectly claiming randomization, or control group, or blinding, or type of animals.

If no: what was the inaccuracy: randomization, blinding, intervention, other.

Abstract: accurate. Does the abstract include mention of some background, objectives (including species or strain), methods, results, and conclusions; and is this mention accurate. Examples: discrepancy between abstract and text; or conclusions not supported by text.

If no, what was the omission (missing background, objectives, methods, results, conclusions) or inaccuracy (difference between abstract and text in any of above areas).

Introduction:

Objectives and/or hypothesis in PICO format:

-Objectives: questions (broad concepts) that the study was designed to answer.

-Hypotheses: pre-specified questions being tested to help meet the objectives. More specific, and amenable to explicit statistical evaluation, than objectives.

-PICO: mention of patients (type of animal), interventions used, comparator (the control group, may be placebo, or usual care, or sham surgery), and outcome (may be mortality, functional outcome, histology, physiology, etc). The objectives and/or hypotheses should mention these four factors, or they should be obvious from the context of the introduction.

-Superiority trial: null hypothesis is that the treatments are equally effective with respect to the primary outcome.

Methods:

Total number of animals used in the experiment. This is from reading methods and results section.

-stated in methods section: Yes, No, Incomplete

-If yes or incomplete:

Number stated _________

Number not stated: <5, >5, Unclear

-any extra in the results section: are any extra animals used obvious from reading the results section (numbers are different from methods, and higher numbers were used than claimed in the methods section). If the number of animals used is not stated in the methods section, then by definition we will answer "yes" to any extra animals in the results section. If yes:

-how many more: exact number or unclear. If unclear, then the choices are: <5, ≥5, or estimated minimum (and give this minimum number).

-how many animals in the largest experimental group: number __________ in the largest group.

Randomization: assigned (allocated) to groups on the basis of chance, at random, in a way that cannot be predicted.

-Non-randomized methods of allocation: alternation, dates, picked 'randomly' out of the cage, historical controls (animals that had similar care without the intervention at an earlier time), other. These are not random (are deterministic allocation).

If randomized: the details:
-Randomization procedure: how the sequence of randomization was generated (an unpredictable allocation sequence). Examples: random number table, computerized random number generator, other.
-Block randomization: to ensure close balance of numbers at any time during the study, can randomize a set number of animals repeatedly. For example, can randomize every x number of animals repeatedly.
-Stratified randomization: to ensure good balance of animal characteristics in each group in small trials. Separate randomization procedure within each subset of animals, such as weight, age, sex, etc.
-Factorial: two or more interventions are sequentially randomized, to assess combinations of treatments.
-Allocation concealment: concealment of the allocation sequence from those enrolling animals until assignment to the group occurs. Methods include- a third-party tells what group that animal goes to, sealed sequentially numbered opaque containers/envelopes.

Blinding: also called masking. The group the animal is in is not known by the humans involved during the experiment. Need to describe who is blinded:

-disease induction: those inducing the disease (eg. injecting the bacteria). If the disease induction occurs before randomization, then it is not applicable (N/A), and if the other two points are not blinded, the answer is "no" for blinding.
-intervention/treatment: those carrying out the intervention (eg. giving antibiotics, drug, or surgical treatment) and caring for the animals during the experiment. For some studies where there are several experiments done, there may be blinding for only some of the treatments/interventions.
-outcome assessment: those collecting the data or scoring the outcome. Particularly important for subjective outcomes (scoring system, histology or pathology, etc). Objective outcomes are those that are not open to interpretation: death or survival; a measured physiologic variable at a specific time; etc.

Experimental unit: the study unit that is allocated to receive the intervention or not. Can be individual animal, or groups of animals.

-parallel group trial: the experimental unit is each animal.
-cluster trial: the experimental unit is a group of animals (eg. a lab, cage, litter).

Eligibility criteria: should be stated prior to starting the experiment, using any word in italics below-
-method of *recruitment* of animals: from where are the animals chosen.
-*inclusion*: what animals were specified to be included in the study.
-*exclusion*: what animals were specified to be excluded from the study.

Study procedures:
Sepsis model: a study to test a treatment for sepsis. The sepsis (infection) is induced in the animal model, using- injection or exposure to a bacterium, virus, parasite, or fungus; injection or exposure to a sepsis mediator (cytokine, chemokine, bacterial product or antigen, other micro-organism product or antigen); creation of an infection by bowel puncture; an infection model such as of pneumonia, peritonitis (abdominal infection), urinary infection, meningitis, wound infection, fascitis, abscess, bacteremia, etc.

-supportive therapy: in humans, treatments include fluid given intravenously (in animals it may be subcutaneously also), antibiotics, surgery, and more. To be relevant to humans it would be best to include at least giving fluid and antibiotics.
-previous underlying illness: in humans, sepsis often complicates an underlying disease, such as chronic lung disease, heart disease, stroke, liver disease, cancer, etc. All of these can modify the response to treatment. To be more relevant to humans it would be best to see if these factors modify the response.
-model used: most human infections are due to a focal infection (a site of infection other than bacteremia, see above), receive the intervention after sepsis occurs (post-treatment), and do not die in the first 24 hours of infection. Many animal models are intravenous bolus models, with the intervention given prior to sepsis (pre-treatment), and death in the first 6-24 hours; these may all be less relevant to humans.

Preparation: how the animals were prepared before the study started.

-Acclimation: time to get used to the environment of the lab.
-Habituation: time to get used to the procedures used in the study, such as restraint, blood-taking, etc.
-Quarantine: time in isolation to be sure no infection is present.

Staff:
-how many laboratory staff involved in the study
-any training for induction of disease, giving intervention, monitoring and measuring outcomes.
-mentioning a veterinarian is involved for some aspect is not enough to qualify as description of staff, unless every aspect of the study animal care was done by the veterinarian.

Animal description: any mention of the following (in either methods or results section)
Species: examples are rat, mouse, pig, dog, cat, etc.
Strain: any mention of strain below species level.
Sex: not simply saying "both sexes". If both sexes, need numbers of each to be stated.
Age
Developmental stage: an explicit reference to whether the animals used are all neonatal, or juvenile (child equivalent), or adults.
Weight
Strain, sex, and either weight or age
Source: specify if local or commercially acquired.

-colony

-transport to lab
-previous use of those animals

Genotype (examples: knockout, transgenic, other): need to specifically state the genotype of all (not just say: "mixed breeds").

Statistical methods:

Sample size calculation: should describe the primary outcome, significance level (p value, alpha value; the probability of a type I error, that is, rejecting the null hypothesis when the null hypothesis was true, a false positive finding), power (1-ß; the probability of a type II error, that is, accepting the null hypothesis when the null hypothesis is false, a false negative finding), baseline risk (the risk for the primary outcome in the control group; and standard deviation if a continuous outcome), minimally important difference (the size of a change in the primary outcome which would be important to detect).

-if cluster trial (groups of animals are allocated to the two experimental groups, eg, allocated by cage, litter, laboratory, etc): sample size needs to be adjusted for cluster effect if the outcomes are measured at the individual animal level (i.e. outcomes not measured at the aggregated cluster level). There is non-independence of the outcome and exposures within a group of animals that are housed together or have something more in common. Need *cluster size*, *intracluster (intraclass) correlation coefficient* (the degree of correlation within clusters; the proportion of total variance of the outcome that can be explained by the variation between clusters), to calculate a *design effect*, which is multiplied by the sample size to calculate the required number of clusters. Any mention of one of the italicized words above will be considered adjusting the sample size for cluster design.

-If sample size calculation is done, then determine if the following are reported: the minimally important difference (the difference between groups that the study is to be powered to detect), alpha (the p-value), and beta/power.
-statistical method for analysis is specified before experiment starts.

Primary outcome: used for sample size calculation, and the main outcome of interest that will be statistically tested. This can be a composite outcome (i.e. one outcome that is combined other outcomes: eg. survival with good neurological status is a single composite outcome).
-pre-specified: should be specified before the experiment starts.
-number of primary outcomes: usually should be one.

Secondary outcome(s): other outcomes that are analyzed in the study.
-pre-specified: should be specified before the experiment starts. We will accept anything described in the methods as being measured, and that can be used as an outcome in the study. We will not require explicitly stating "the following are secondary outcomes".
-number of secondary outcomes: often there are many. Categorized as none, <5, 5-10, >10.
-clearly defined: not open to later interpretation; it is clear how the outcome will be measured.
-statistical method for analysis for each is specified before the experiment starts.

Subgroups: to look for evidence of a difference in treatment effect in complementary subgroups.

-pre-specified: should be specified before the experiment starts
-how many: this refers to how many subgroup analyses are specifically described in the methods section (i.e. pre-planned subgroup analyses). There are often several.

Results:
Baseline characteristics: should be reported to allow readers to determine if there is any prognostic imbalance between the groups. Best way to present this is in a table.
-demographics: age, sex, weight, microbiologic status, drug/test naïve, etc
-clinical: physiology (blood pressure, heart rate, temperature, respiratory rate)

Primary outcome:
-numbers analyzed clearly stated: denominators clear (not just a percentage given; absolute numbers are explicitly stated, and not implied so that it takes reading at several places in the manuscript to figure out the numbers exactly).
-numbers analyzed same as in methods section: no unaccounted for animals not in analysis.
-any excluded from analysis:
    -numbers stated
    -reasons for any exclusions from analysis stated
-numbers clear in most (the majority, over 50%) tables and graphs
-all pre-specified primary outcomes reported
    -if not: how many not reported.
-measure of precision for outcome: report the outcome with a 95% confidence interval, standard deviation, or standard error, in addition to the p-values.
-absolute difference, and relative difference reported: the relative effect can be reported as risk ratio, odds ratio, risk difference, hazard ratio.
-positive and negative outcomes:
    -number of positive primary outcomes: _______. Positive outcome is a better outcome with the studied intervention (in direction that suggests benefit from intervention or in direction hypothesized before the study started).
    -number of neutral or negative primary outcomes:_______. Negative outcome is a worse outcome with the studied intervention (in direction that suggests harm from intervention, or is opposite to what was hypothesized before the study). Neutral outcome is no difference in outcome between control and intervention group.
-intention to treat analysis: this is a misused term. For our purposes, this means that all animals that underwent randomization and have an outcome measured are analyzed in the group to which they were originally allocated. Since some animals may have missing outcomes, this is really a 'complete available case analysis' or 'full analysis set'.
    Some may be excluded, if they did not meet pre-specified eligibility criteria, received a treatment not allowed by the protocol, did not take all of the intended treatment, received the wrong treatment or no treatment by error. This exclusion after allocation is called attrition, may be unavoidable, but an analysis that excludes these animals is called 'modified intention to treat', 'per protocol', or 'on treatment' analysis and is not the same as 'intention to treat'.
-any subgroup analyses: were they pre-specified in the methods, or post-hoc. And were they tested by a 'test of interaction'. This means, not just looking at comparing p-values between subgroups; rather, reporting the difference in intervention effect in each subgroup (will mention at least one of the following italicized words: an *interaction term*, *cross-product term* used to assess the presence of '*effect modification*' by subgroup).

Secondary outcomes: as applied to the majority of secondary outcomes.
-numbers analyzed clearly stated for most (the majority, over 50%): denominators clear (not just a percentage given).
-numbers analyzed same as in methods section: no unaccounted for animals not in analysis.
-any excluded from analysis:

  -numbers stated: the numbers excluded from most analyses should be clearly stated.

  -reasons for any exclusions from analysis clearly stated

-numbers clear in most (the majority, over 50%) tables and graphs
-all pre-specified important secondary outcomes reported: not reporting or clearly reporting a minor outcome will not be recorded. This response applies to omitting a major outcome that is clearly relevant to the study objectives.

  Terminology: these tests can be considered to include any of the following. This is intended to be 'generous', meaning, if they report any of these outcomes from the test, it will considered pre-specified.

  -Arterial blood gas (ABG): can include Pa02, PaC02, HCO3 (bicarbonate), pH, electrolyes (Na, K, Cl), lactate.

  -Complete blood count (CBC): can include white blood cell count (WBC), hemoglobin, hematocrit, platelet count.

  -Liver function tests: can include alanine transaminase (ALT), aspartate transferase (AST), alkaline phosphatase (Alk Phos), bilirubin.

  -Kidney function tests: can include urea, and creatinine. If urine is collected also, they can calculate the creatinine clearance.

-measure of precision for outcome: report the outcome with a 95% confidence interval, standard deviation, or standard error, in addition to the p-values. The Yes or No applies to the majority of secondary outcomes. If yes, the choice of CI, SD, SE, applies to any use of each of these (not necessarily in the majority of outcomes for each).
-absolute difference, and relative difference reported: the relative effect can be reported as risk ratio, odds ratio, risk difference, hazard ratio.
-number of outcomes:

  -mostly positive outcomes: Yes or No. Positive outcome is a better outcome with the studied intervention (in direction that suggests benefit from intervention).

  -any negative outcomes: Yes or No. Negative outcome is a worse outcome with the studied intervention (in direction that suggests harm from intervention). To detect these, we will only include a negative outcome that is explicitly mentioned as negative (eg. wording as- adverse, worse, negative, harmful, etc) or is obviously negative considering the studies objectives.

  -number of posthoc outcomes: none, <5, 5-10, >10. Any secondary outcomes that were not pre-specified in the methods, and therefore were done post-hoc (post-hoc means after looking at the data, and these analyses are prone to false positive findings).

  -number of statistical comparisons performed: <5, 5-20, 21-40, >40. Some studies analyze dozens of outcomes. We will report the total number of outcomes analyzed (number of statistical comparisons performed) by multiplying the number of outcomes by the number of groups compared for each of these outcomes. Note that in some studies several groups are compared using ANOVA, and then if the difference is significant, subtests are done to see where the difference is (i.e. between which groups); in this case, the comparison between all the groups is considered one statistical test.

-any subgroup analyses: were they pre-specified in the methods, or post-hoc. And were they tested by a 'test of interaction'. This means, not just looking at comparing p-values between subgroups; rather, reporting the difference in intervention effect in each subgroup (will mention at least one of the italicized terms: an *interaction term*, *cross-product term* used to assess the presence of '*effect modification*' by subgroup).

-subgroup: looking for a difference in intervention effect in complementary subgroups of animals. This can refer to a demographic (eg. male vs female; old vs young; etc), disease (eg. infected vs noninfected; hypertensive vs nonhypertensive; etc), physiologic (eg. high vs low heart rate; high vs low tidal volume; high vs low inspiratory time; etc); or other complementary subgroups that were not what the randomization was based upon.

-Toxicity reported: abnormal laboratory measurements are drug related harms, or toxicity. If mention any occurring, or explicitly state none occurred, then toxicity is reported. If no drug involved, then answer is N/A.

Discussion:

-internal validity limitations discussed: any mention of- multiple comparisons leading to risk of false findings, sample size too small to detect a difference, biases that may be present [*selection bias* due to lack of randomization or allocation concealment, *performance bias* due to lack of blinding by carers, *ascertainment bias* due to lack of blinding by outcome assessors, *attrition bias* due to unequal exclusions after allocation or lack of ITT analysis).

-external validity (generalizability) discussed: the extent to which the results can be generalized to other circumstances, depending on the comparability between the study animals and the population of interest.

Generalization to humans discussed: any mention of possible use of the data to apply to humans. If so, is there mention of any limitation in applying this to humans: examples include mention of differences in severity, co-morbidity (other disease states such as high blood pressure, diabetes, heart disease, etc), co-interventions (other care provided such as hospitalization, intensive care, fluids, drugs, etc), timing of interventions (feasibility of timing of intervention), outcomes assessed (other outcomes more important to humans, such as function, quality of life, late outcomes weeks or months later, etc). If only mention a vague statement that animal data may not translate to humans, without any mention of reasons why this is the case, then answer is "vague".

-funding: source described. If described, was it a government source (NIH= national institutes of health, CIHR= Canadian Institutes of Health Research, etc), charity or foundation (HSF= heart and stroke foundation, Diabetes association, lung association, etc), industry (drug company, other development company or manufacturer), or other. -Did the funder have any role in study analysis or reporting of results: yes, no, or not reported. A statement that there was an "unrestricted" grant will be considered as a 'no'.
