# Supplementary material for: The methodological quality of animal research in critical care: the public face of science
Source: Ann Intensive Care. 2014 Jul 29;4:26. doi: 10.1186/s13613-014-0026-8 (PMC4126494; doi:10.1186/s13613-014-0026-8)
Supplement: Additional file 2: — Case Report Form for Methodological Quality of animal research study. This file shows the case report form used to abstract the variables from each animal research study, according to the definitions given in the manual for the case report form. [file s13613-014-0026-8-S2.pdf]

Additional File 2: Case Report Form for Methodological Quality of animal research study.

Authors: Meredith Bara BSc and Ari R Joffe MD, FRCPC

Title:____

____

Journal: CCM, ICM, AJRCCM
Year; Volume: page numbers:____

**Variable**	**Data**	**Comments**
**Title**: accurate and concise	Yes or No	If no: randomization; blinding; intervention; other.
**Abstract**: accurate	Yes or No	If no:<br>-Omission: background, objectives, methods, results, conclusions<br>-Inaccuracy: background, objectives, methods, results, conclusions.
**Introduction:**	-	-
Objectives/hypotheses in PICO format	Yes or No	-
**Number of animals used**	-	-
Stated in methods section	____________	-
Any extra in results section	Yes or No	If yes: answer bullets below
-how many more	___________	If number not clear: <5, 5 or more, unclear.
Number in the largest group	____________	-
**Methods:**	-	-
Design:	-	-
Randomized	Yes or No	If no: alternation; dates; picked out of cage; historical control; not specified, other___________<br>If yes: answer the bullets below
-Procedure described	Yes or No	Table; computer; other ___________
-Block randomization	Yes or No	If yes: size of blocks: ____
-Stratified randomization	Yes or No	If yes: number of characteristics _____
-Factorial	Yes or No	If yes: number of factors ______
-Allocation concealment	Yes or No	If yes: third party; envelopes; other ___________
Blinding	Yes or No	If yes: answer the bullets below.
-disease induction	Yes, No, N/A, or Unclear	-
-treatment/intervention	Yes, No, Some, or Unclear	-
-outcome assessment	All, Some, or None	If none or some: any subjective outcomes not blinded- Yes or No
Experimental Unit	Yes or No	Group; Individual; Part of individual
Eligibility criteria	Yes or No	-
Study procedures:	-	-
Sepsis model	Yes or No	If yes: answer the bullets below.

-supportive therapy	Yes or No	If yes: fluids; antibiotics; other
-intercurrent illnesses	Yes or No	If yes: describe_________________
-model relevance	-	Focal or bolus; ≤ or > 24 hours; treatment intervention is pre- or post-sepsis induction
Preparation prior to experiment	Yes or No	If yes: acclimation; habituation; quarantine
Staff description	Yes or No	If yes: number; training in induction; intervention; monitoring; outcome
Animal description:	-	-
Species	Yes or No	_______________________
Strain	Yes or No	-
Sex	Yes or No	-
Age	Yes or No	-
Developmental stage	Yes or No	If yes: neonate; juvenile; adult
Weight	Yes or No	-
Strain, sex, and either weight or age	Yes or No	-
Source	Yes or No	If yes: commercial or local; colony; transport; previous use
Genotype	Yes or No	-
**Statistics:**	-	-
Sample size calculated	Yes or No	If cluster trial: cluster adjustment YorN<br>If sample size calculated: explicit mention of alpha, beta/power, minimally important difference.
Primary outcome specified	Yes or No	If yes: answer the bullets below.
-number of primary outcomes	-	Number _________
-statistical method specified	Yes or No	-
Secondary outcomes specified	Yes or No	If yes: answer the bullets below.
-number of secondary outcomes	-	Number: none, <5, 5-10, >10.
-statistical methods specified	Yes or No	-
Sub-groups to be analyzed?	Yes or N/A	If yes: answer the bullets below.
-how many?	-	Number____________
**Results:**	-	-
baseline characteristics compared in table	Yes or No	If yes: answer the bullets below.
-at least two demographics	Yes or No	
-at least two physiology	Yes or No	
Primary outcome analyzed	Yes or No or N/A	If yes: answer the bullets below.
-numbers with denominators	Yes or No	-
-unaccounted numbers	Yes, No, or Unclear	-
-any excluded from analysis	Yes or No or Unclear	If yes: number stated; reasons stated

-numbers in tables/graphs	Yes or No	-
-all pre-specified reported	Yes or No	If no: number not reported______
-measure of precision	Yes or No	If yes: CI; SD; SE
-absolute and relative effect	Yes or No	-
-number positive outcome	-	Number____
-number negative or neutral outcome	-	Number____
-intention to treat analysis	Yes or No	-
-pre-specified subgroups analyzed	Yes or No or N/A	If yes: test of interaction done YorN
-post-hoc subgroups analyzed	Yes or N/A	If yes: number_________; test of interaction done YorN
Secondary outcomes analyzed	Yes or No	If yes: answer the bullets below as they apply to the majority of the outcomes.
-numbers with denominators for most	Yes or No	-
-unaccounted numbers	Yes or No or Unclear	-
-any excluded from analysis	Yes or No or Unclear	If yes: number stated; reasons stated
-numbers in most tables/graphs	Yes or No	-
-all pre-specified important outcomes reported	Yes or No	-
-measure of precision	Yes or No	If yes: CI; SD; SE
-absolute and relative effect	Yes or No	-
-mostly positive outcomes	Yes or No	-
-any negative outcome (obvious, or explicitly stated)	Yes or No	-
-number post-hoc outcomes (not pre-specified in methods)	-	Number: none; <5; 5-10; >10
-number of statistical comparisons	-	<5; 5-20; 21-40; >40
-pre-specified subgroups analyzed	Yes or No or N/A	If yes: number_______; test of interaction done YorN
-post-hoc subgroups analyzed	Yes or N/A	If yes: number_______; test of interaction done YorN.
Any toxicity or lack of toxicity mentioned or reported	Yes, No, or N/A	-
**Discussion:**	-	-
Internal validity: limitations discussed	Yes or No	If yes: sample size; bias; multiple comparisons.
External validity: to humans discussed	Yes or No	If yes: any limitation to generalizing to humans mentioned- Yes or No or Vague
Funding source described	Yes or No	If yes: answer the bullets below.
-government source	Yes or No	-

-foundation charity	Yes or No	-
-industry company	Yes or No	-
-any role in analysis or reporting	Yes or No or NR	-
**Any Comments:**		

Notes:
-circle correct response for Yes or No
-circle all that apply for sub-questions in last column
-fill in underlined space as applicable with numbers or names
