# Supplementary material for: The methodological quality of animal research in critical care: the public face of science
Source: Ann Intensive Care. 2014 Jul 29;4:26. doi: 10.1186/s13613-014-0026-8 (PMC4126494; doi:10.1186/s13613-014-0026-8)
Supplement: Additional file 3: — The publications included in the Methodological Quality of animal research study. This file includes the list of the 77 publications reviewed for this study. [file s13613-014-0026-8-S3.pdf]

Additional File 3: The publications included in the Methodological Quality of animal research study.

Authors: Meredith Bara BSc and Ari R Joffe MD, FRCPC.

References for the 77 animal research publications reviewed for methodological quality in three critical care journals during 6 months (Jan-June) of the year 2012.

Critical Care Medicine:

Hu H, Xenocostas A, Chin-Yee N, et al. Transfusion of fresh but not old stored blood reduces infarct size and improves cardiac function after acute myocardial infarction in anemic rats.
CCM 2012, 40, 740-746

Pantazopoulos IN, Xanthos TT, Vlachos I, et al. Use of the impedance threshold device improves survival rate and neurological outcome in a swine model of asphyxial cardiac arrest
CCM 2012, 40, 861-868

Pecchiari M, Ario M, Antonia K, et al. Plasma membrane disruptions with different models of injurious mechanical ventilation in normal rat lungs CCM 2012, 40, 869-875

Benavides U, Gonzalez-Murguiondo M, Harii N, et al. Phenylmethimazole inhibits production of proinflammatory mediators and is protective in an experimental model of endotoxic shock CCM 2012, 40, 886-894

Sheng C, Yu YH, Zhao KS, et al. Acute lung inflammatory response and injury after hemorrhagic shock are more severe in postpartum rabbits CCM 2012, 40, 1570-1577

Yoshida T, Uchiyama A, Matsuura N, et al. Spontaneous breathing during lung-protective ventilation in an experimental acute lung injury model: High transpulmonary pressure associated with strong spontaneous breathing effort may worsen lung injury.
CCM 2012, 40, 1578-1585

Yannopoulos D, Segal N, McKnite S, et al. Controlled pauses at the initiation of sodium nitroprusside enhanced cardiopulmonary resuscitation facilitate neurological and cardiac recovery after 15 minutes of untreated ventricular fibrillation CCM 2012, 40, 1562-1569

Li Bassi G, Saucedo L, Joan-Daniel M, et al. Effects of duty cycle and positive end expiratory pressure on mucus clearance during mechanical ventilation CCM 2012, 40, 895-902

Muders T, Leupschen H, Jorg Z, et al. Tidal recruitment assessed by electrical impedance tomography and computed tomography in a porcine model of lung injury CCM 2012, 40, 903-911

Leon K, Pichavant-Rafini K, Quemener E, et al. Oxygen blood transport during experimental sepsis: Effect of hypothermia CCM 2012, 40, 912-918

Beurskens CJP, Aslami H, Kuipers MT, et al. Induced hypothermia is protective in a rat model of pneumococcal pneumonia associated with increased adenosine triphosphate availability and turnover CCM 2012, 40, 919-926

Smuder AJ, Hudson MB, Nelson WB, ete al. Nuclear factor kB signaling contributes to mechanical ventilation-induced diaphragm weakness CCM 2012, 40, 927-934

Timaru-Kast R, Wyschkon S, Luh C, et al. Delayed inhibition of angiotensin II receptor type I reduces secondary brain damage and improves functional recovery after experimental brain injury CCM 2012, 40, 935-944

Bitto A, Polito F, Irrera N, et al. Protective effects of melanocortins on short term changes in a rat model of traumatic brain injury CCM 2012, 40, 945-951

Esen F, Senturk E, Ozcan PE, et al. Intravenous immunoglobins prevent the breakdown of the blood brain barrier in experimentally induced sepsis CCM 2012, 40, 1214-1220

Empey PE, Miller TM, Philbrick AH, et al. Mild hypothermia decreases fentanyl and midazolam steady state clearance in a rat model of cardiac arrest CCM 2012, 40, 1221-1228

Bouchama A, Al-Mohanna F, Assad L, et al. Tissue factor/factor VIIa pathway mediates coagulation activation in induced-heat stroke in the baboon CCM 2012, 40, 1229-1236

Gill RS, Manouchehri N, Liu JQ, et al. Cyclosporine treatment improves cardiac function and systemic hemodynamics during resuscitation in a newborn piglet model of asphyxia: A dose-response study CCM 2012, 40, 1237-1244

Chien MS, Bien MY, Ku CC, et al. Systemic human orbital fat-derived stem/stromal cell transplantation ameliorates acute inflammation in lipopolysaccharide-induced acute lung injury CCM 2012, 40, 1245-1253

Hudson MB, Smuder AJ, Nelson WB, et al. Both high level pressure support ventilation and controlled mechanical ventilation induce diaphragm dysfunction and atrophy CCM 2012, 40, 1254-1260

Chan YL, Orie NN, Dyson A, et al. Inhibition of vascular adenosine triphosphate-sensitive potassium channels by sympathetic tone during sepsis CCM 2012, 40, 1261-1268

Boros M, Ghyczy M, Erces D, et al. The anti-inflammatory effects of methane. CCM 2012, 40, 1269-1278

Sun CK, Chang CL, Lin YC, et al. Systemic administration of autologous adipose-derived mesenchymal stem cells alleviates hepatic ischemia-reperfusion injury in rats CCM 2012, 40, 1279-1290

Zhan Y, Chen C, Suzuki H, et al. Hydrogen gas ameliorates oxidative stress in early brain injury after subarachnoid hemorrhage in rats CCM 2012, 40, 1291-1296

Fries M, Brucken A, Cizen A, et al. Combining xenon and mild therapeutic hypothermia preserves neurological function after prolonged cardiac arrest in pigs CCM 2012, 40, 1297-1303

Zhuang L, Yang T, Zhao H, et al. The protective profile of argon, helium and xenon in a model of neonatal asphyxia in rats
CCM 2012, 40, 1724-1730

Metzger AK, Herman M, McKnite S, et al. Improved cerebral perfusion pressures and 24hr neurological survival in a porcine model of cardiac arrest with active compression-decompression CPR and aug of intrathoracic pressure CCM 2012, 40, 1851-1856

Nelson WB, Smuder AJ, Hudson M, et al. Cross talk between calpain and caspase-3 proteolytic systems in the diaphragm during prolonged mechanical ventilation CCM 2012, 40, 1857-1863

Chierichetti M, Engelberts D, El-Khuffash A, et al. Continuous negative abdominal distension augments recruitment of atelectatic lung
CCM 2012, 40, 1864-1872

Regli A, Chakera J, De Keulenaer BL, et al. Matching positive end expiratory pressure to intra-abdominal pressure prevents end-expiratiory lung volume decline in a pig model of intra-abdominal hypertension CCM 2012, 40, 1879-1886

Kuper C, Fraek ML, Muller HH, et al. Sepsis-induced urinary concentration defect is related to nitric oxide dependent activation of TonEBP/NFAT5, which downregulates renal medullary solute transport proteins and aqua porin 2. CCM 2012, 40, 1887-1895

Smeding L, Leong-Poi H, Hu P, et al. Salutary effect of resveratrol on sepsis-induced myocardial depression. CCM 2012, 40, 1896-1907

Altay O, Hasegawa Y, Sherchan P, et al. Isoflurane delays the development of early brain injury after subarachnoid hemorrhage thru sphingosine-related pathway activation in mice. CCM 2012, 40, 1908-1913

Lee BF, Wang LW, Lin SH, et al. Tc-99m-HL91 imaging in the early detection of neuronal injury in a neonatal rat model of hypoxic ischemia. CCM 2012, 40, 1930-1938

Rehberg S, Enkhbaatar P, Rehberg J, et al. Unlike arginine vasopressin, the selective Via receptor agonist FE 202158 does not cause procoagulant effects by releasing von Willebrand factor. CCM 2012, 40, 1957-1960

Martinez-Olondris P, Rigol M, Soy D, et al. Efficacy of linezolid compared to vancomycin in an experimental model of pneumonia induced by methicillin-resistant Staphylococcus aureus in ventilated pigs. CCM 2012, 40, 162-168

Filseth OM, How OJ, Kondratiev T, et al. Changes in cardiovascular effects of dopamine in response to graded hypothermia. CCM 2012, 40, 178-186

Pejo E, Feng Y, Chao W, et al. Differential effects of etomidate and its pyrrole analogue carboetomidate on the adrenocortical and cytokine responses to endotoxemia. CCM 2012, 40, 187-192

Heckel K, Winkelmann B, Struden MS, et al. Tetrastarch sustains pulmonary microvascular perfusion and gas exchange during systemic inflammation. CCM 2012, 40, 518-531

Hicks CW, Sweeney DA, Danner RL, et al. Efficacy of selective mineralocorticoid and glucocorticoid agonists in canine septic shock
CCM 2012, 40, 199-207

Moinard C, Barbar S, Choisy C, et al. Arginine reduces bacterial invasion in rats with head injury: An in vivo evaluation by bioluminescence. CCM 2012, 40, 278-280

Cabrera-Benitez NE, Parotto M, Post M, et al. Mechanical stress induces lung fibrosis by epithelia-mesenchymal transition. CCM 2012, 40, 510-517

Yabluchanskiy A, Sawle P, Homer-Vanniasinkam S, et al. CORM-3, a carbon monoxide-releasing molecule alters the inflammatory response and reduces brain damage in a rat model of hemorrhagic stroke. CCM 2012, 40, 544-552

Chen TY, Lin MHS, Lee WT, et al. Nicotinamide inhibits nuclear factor - kappa B translocation after transient focal cerebral ischemia. CCM 2012, 40, 532-537

Peng ZY, Wang HZ, Srisawat N, et al. Bactericidal antibiotics temporarily increase inflammation and worsen acute kidney injury in experimental sepsis. CCM 2012, 40, 538-543

Li Y, Ristagno G, Guan J, et al. Preserved heart rate variability during therapeutic hypothermia correlated to 96 hrs neurological outcomes and survival in a pig model of cardiac arrest. CCM 2012, 40, 580-586

Maybhate A, Hu C, Bazley FA, et al. Potential long term benefits of acute hypothermia

after spinal cord injury: Assessments with somatosensory-evoked potentials. CCM 2012, 40, 573-579

Gotes J, Kasian K, Jacobs H, et al. Benefits of ethyl gallate versus norepinephrine in the treatment of cardiovascular collapse in Pseudomonas aeruginosa septic shock in dogs. CCM 2012, 40, 560-572

Wu SY, Wu CP, Kang BH, et al. Hypercapnic acidosis attenuates reperfusion injury in isolated and perfused rat lungs. CCM 2012, 40, 553-559

Yeh KH, Sheu JJ, Lin YC, et al. Benefit of combined extracorporeal shockwave and bone marrow-derived endothelial progenitor cells in protection against critical limb ischemia in rats. CCM 2012, 40, 169-177

Intensive Care Medicine:

Waisman D, Faingersh A, Levy C, et al. Early detection of deteriorating ventilation by monitoring bilateral chest wall dynamics in the rabbit. ICM 2012, 38, 120-127

Shah KG, Wu R, Jacob A, et al. Recombinant human milk fat globule-EGF factor 8 produces dose-dependent benefits in sepsis. ICM 2012, 38, 128-136

Zlotnik A, Leibowitz A, Gurevich B, et al. Effect of estrogens on blood glutamate levels in relation to neurological outcome after TBI in male rats. ICM 2012, 38, 137-144

Bhatia R, Schmolzer GM, Davis PG, et al. Electrical impedance tomography can rapidly detect small pneumothoraces in surfactant-depleted piglets. ICM 2012, 38, 308-315

Chakkarapani E, Thoresen M, Liu X, et al. Xenon offers stable haemodynamics independent of induced hypothermia after hypoxia-ischaemia in newborn pigs. ICM 2012, 38, 316-323

Finney SJ, Leaver SK, Evans TW, et al. Differences in lipopolysaccharide and lipoteichoic acid-induced cytokine/chemokine expression. ICM 2012, 38, 324-332

Cheung DC, Gill RS, Liu JQ, et al. Vasopressin improves systemic hemodynamics without compromising mesenteric perfusion in the resuscitation of asphyxiated new born piglets: a dose-response study. ICM 2012, 38, 491-498

Santos CL, Moraes L, Santos RS, et al. Effects of different tidal volumes in pulmonary + extrapulmonary lung injury with or without intraabdominal hypertension. ICM 2012, 38, 499-508

Nilsson MCA, Freden F, Larsson A, et al. Hypercapnic acidosis transiently weakens hypoxic pulmonary vasoconstriction without affecting endogenous pulmonary nitric oxide. ICM 2012, 38, 509-517

Zanella A, Cressoni M, Epp M, et al. Effects of tracheal orientation on the development of ventilator associated pneumonia: an experimental study. ICM 2012, 38, 677-685

Langer T, Carlesso E, Protti A, et al. In vivo conditioning of acid-base equilibrium by crystalloid solutions: an experimental study on pigs. ICM 2012, 38, 686-693

Zambelli V, Di Grigoli G, Scanziani M, et al. Time course of metabolic activity and cellular infiltration in a murine model of acid-induced lung injury. ICM 2012, 38, 694-701

van Hees HWH, Schellekens WJM, Acuna GLA, et al. Titin and diaphragm dysfunction in mechanically ventilated rats. ICM 2012, 38, 702-709

Engelberts D, Malhotra A, Butler JP, et al. Relative effects of negative vs. positive PV depend on applied conditions. ICM 2012, 38, 879-885

May CN, Ishikawa K, Wan L, et al. Renal bioenergetics during early gram-negative mammalian sepsis and angiotensin II infusion. ICM 2012, 38, 886-893

Oyaizu T, Fung SY, Shiozaki A, et al. Src tyrosine kinase inhibition prevents pulmonary ischemia-reperfusion induced acute lung injury. ICM 2012, 38, 894-905

Derive M, Bouazza Y, Alauzet C, et al. Myeloid-derived suppressor cells control microbial sepsis. ICM 2012, 38, 1040-1049

Lourenco AP, Vasques-Novoa F, Oliveira-Pinto J, et al. Haemodynamic and neuroendocrine effects of tezosentan in chronic experimental pulmonary hypertension. ICM 2012, 38, 1050-1060

American Journal of Respiratory and Critical Care Medicine:

Su G, Atakilit A, Li JT, et al. Absence of integrin avB3 enhances vascular leak in mice by inhibiting endothelial cortical action formation. AJRCCM 2012, 185, 58-66

Samapati R, Yang Y, Yin J, et al. Lung endothelial Ca2+ and permeability response to platelet-activating factor is mediated by acid sphingomyelinase and transient receptor potential classical 6. AJRCCM 2012, 185, 160-170

Herzig DS, Driver BR, Fang G, et al. Regulation of lymphocyte trafficking by CXC chemokine receptor 3 during septic shock
AJRCCM 2012, 185, 291-300

Apostolou E, Stavropoulos A, Soundtoulidis A, et al. Activin-A overexpression in the murine lung causes pathology that stimulates ARDS
AJRCCM 2012, 185, 382-391

Grommes J, Alard JE, Drechsler M, et al. Disruption of platelet derived chemokine heteromers prevent neutrophil extravasation in ALI. AJRCCM 2012, 185, 628-636

Zafrani L, Gerotziafas G, Byrnes C, et al. Calpastatin controls polymicrobial sepsis by limiting procoagulant microparticle relsease
AJRCCM 2012, 185, 744-755

Files DC, D'Alessio FR, Johnston LF, et al. A critical role for muscle ring finger 1 in ALI associated skeletal muscle wasting
AJRCCM 2012, 185, 825-835

MacGarvey NC, Suliman HB, Bartz RR, et al. Activation of mitochondrial biogenesis by heme oxygenase-1-mediated NF-E2 related factor-2 induction rescues mice from lethal S. aureus sepsis. AJRCCM 2012, 185, 851-861

Dolinay T, Kim YS, Howrylak J, et al. Inflammasome-regulated cytokines are critical mediators of acute lung injury. AJRCCM 2012, 185, 1225-1234
